# Enhancing the selective extracellular location of a recombinant *E. coli* domain antibody by management of fermentation conditions

**DOI:** 10.1007/s00253-015-6799-3

**Published:** 2015-07-17

**Authors:** Ioannis Voulgaris, Gary Finka, Mark Uden, Mike Hoare

**Affiliations:** BioPharm R&D, BioPharm Process Research, GlaxoSmithKline R&D, Stevenage, SG1 2NY UK; The Advanced Centre for Biochemical Engineering, Department of Biochemical Engineering, University College London, London, WC1H 0AH UK

**Keywords:** Periplasmic release, Polyethyleneimine, Fermentation, Domain antibody, *E. coli*

## Abstract

The preparation of a recombinant protein using *Escherichia coli* often involves a challenging primary recovery sequence. This is due to the inability to secrete the protein to the extracellular space without a significant degree of cell lysis. This results in the release of nucleic acids, leading to a high viscosity, difficulty to clarify, broth and also to contamination with cell materials such as lipopolysaccharides and host cell proteins. In this paper, we present different fermentation strategies to facilitate the recovery of a *V*_H_ domain antibody (13.1 kDa) by directing it selectively to the extracellular space and changing the balance between domain antibody to nucleic acid release. The manipulation of the cell growth rate in order to increase the outer cell membrane permeability gave a small ~1.5-fold improvement in released domain antibody to nucleic acid ratio without overall loss of yield. The introduction during fermentation of release agents such as EDTA gave no improvement in the ratio of released domain antibody to nucleic acid and a loss of overall productivity. The use of polyethyleneimine (PEI) during fermentation was with the aim to (a) permeabilise the outer bacterial membrane to release selectively domain antibody and (b) remove selectively by precipitation nucleic acids released during cell lysis. This strategy resulted in up to ~4-fold increase in the ratio of domain antibody to soluble nucleic acid with no reduction in domain antibody overall titre. In addition, a reduction in host cell protein contamination was achieved and there was no increase in endotoxin levels. Similar results were demonstrated with a range of other antibody products prepared in *E. coli*.

## Introduction

*Escherichia coli* is the most widely used organism for the production of proteins that do not require glycosylation. The well-characterised genome, the ease of cultivation and the rapid growth to high cell densities have contributed to its popularity. As a gram-negative bacterium, *E. coli* can either be used for recombinant protein expression in the cytoplasmic or periplasmic space. Cytoplasmic protein expression usually results in misfolding and aggregation of the heterologous protein into inclusion bodies. Also, the reducing environment of the cytoplasm hinders the formation of disulfide bonds (Baneyx and Mujacic 2004) and there is a need for cell lysis in order to recover the product. Product expression in the periplasmic space needs careful consideration of the method of release from the cell where, for example, the extent of release of protease and other degradative proteins especially from the cytoplasm and long hold times, e.g. to achieve release, can lead to significant loss of protein product (Kaufmann 1997).

Lysis of the cells results in a significant increase in viscosity mainly due to the release of DNA (Balasundaram et al. 2009; Nesbeth et al. 2012) which has a negative effect on the early centrifugation and filtration steps. Release by mechanical processes (Hubbuch et al. 2006; Li et al. 2013) or by use of sonication or focussed acoustics (Li et al. 2012) can result in a reduction of the nucleic acid molecular weight and hence of the viscosity, but this is accompanied by high levels of cell debris attrition leading to difficult solid-liquid separation (Li et al. 2013). Nucleases may be used to degrade the nucleic acids (Garke et al. 2000) or, to avoid the complexity at manufacture scale of added biological agents, the *E. coli* host engineered to co-express a nuclease (Balasundaram et al. 2009). The use of reagents such as polyethyleneimine (PEI) has been shown to selectively flocculate cell debris and precipitate nucleic acids thereby making clarification of homogenised or lysed cells easier (Barany and Szepesszentgyörgyi 2004; Chatel et al. 2014a).

The selective release of periplasmic products has been demonstrated using osmotic shock (Rathore et al. 2003); the use of EDTA to chelate Mg^2+^ and Ca^2+^ and hence, weaken the outer membrane (OM) by destabilising the lipopolysaccharides (LPS) leaflets (Jalalirad 2013); the use of chaotropic agents such as guanidine and urea to weaken the OM resistance (Falconer et al. 1997); the use of lysozyme to digest the peptidoglycan of the cell wall (Pierce et al. 1997). The above are generally applied to the harvested fermentation broth or more selectively on the recovered cells. Challenges faced are of scale up and achieving selective recovery away from cytoplasmic components. The successful release to the extracellular space of a periplasmic recombinant β-lactamase during *E. coli* fermentation has been reported using EDTA and phenethyl alcohol (Ryan and Parulekar 1991).

Directed extracellular secretion of a protein product might be achieved by use of alternative hosts. For example use of yeasts such as *Pichia pastoris* and *Saccharomyces cerevisiae* can yield high expression titres but can cause unwanted glycosylation and product degradation due to proteolysis (Johnson 2013; Potvin et al. 2012). Gram-positive *Corynebacterium glutamicum* has been engineered to remove cell wall proteins which act as permeability barriers to increase antibody fragment secretion albeit at relatively low levels (Matsuda et al. 2014). For *E. coli*, a similar strategy has been used (Yoon et al. 2010) as has (a) the co-expression of proteins that help permeabilise the outer cell membrane such as the bacteriocin release protein (Sommer et al. 2010) and the colicin E1 lysis protein (Mergulhão et al. 2005), (b) the fusion of the target protein with host cell proteins such as OM protein F (Jeong and Lee 2002) or the osmotically inducible protein Y and YebF (Zhang et al. 2006) to promote secretion (here, selective cleavage is needed to remove the secretion vector), (c) the use of *E. coli* mutants lacking genes for structural elements of the OM and peptidoglycan layer (Shokri et al. 2003). Despite the enhanced product leakage demonstrated in all of these cases, these strains are not appropriate for industrial high cell density production since they are growth impaired, they are prone to premature lysis and they lack the necessary robustness (Mergulhão et al. 2005; Shokri et al. 2003).

In this paper, we examine three fermentation-based strategies to enhance the ratio in the extracellular space of domain antibody product release to nucleic acid release. These include the following: (i) the manipulation of carbon feed rate in order to increase the outer cell membrane permeability, (ii) the incorporation of reagents within the broth designed to increase the cell membrane permeability, (iii) the use of PEI to both increase the cell OM permeability and remove selectively released nucleic acids.

## Materials and methods

### Fermentation method

An *E. coli* W3110 (ATCC® 27325TM) strain is used incorporating the pAVE011 plasmid (Hodgson et al. 2007) carrying the gene for a V_H_ anti-TNFR1 domain antibody of 13.1 kDa (see Chatel et al. 2014b for gene sequence) fused to the OmpA leader sequence (accession no. P0A910, Choi and Lee 2004; Lee et al. 1998). The protein produced is from here on noted as domain antibody (dAb). This construct was used for all fermentation studies reported here. Three other *E. coli* constructs, producing different antibody-related molecules, used to evaluate generality of main conclusions are briefly described in the relevant “Results” section.

Inoculum was prepared using 1 ml of glycerol (20 %, *v/v*) stock of the cells (stored at −80 °C) introduced into 400 ml of vLB inoculation medium in a 1000-ml baffled flask (Ultra Yield Flask™ Thomson Instrument Company, Kent, UK) in a rotary-shaking incubator at 220 rpm and 37 °C until OD_600_ reached 1. This was used to inoculate at 1:50 *v/v* eight bioreactors each of 1-l working volume (SR1000DLL bioreactor, vessel diameter 100 mm, aspect ratio 2.4:1, overhead driven triple Rushton 6-blade impellers (46-mm diameter), DASGIP AG, Julich, Germany) charged with complex medium containing glycerol as the main carbon source and yeast extract-soytone. The reactor was maintained at pH 7.0 ± 0.05 using 25 % (*v/v*) NH_4_OH and 2 M H_3_PO_4_ at temperatures of 30 °C before induction and 26 °C after induction; at DO of 30 ± 5 %, by cascade control using firstly impeller speed (400–1600 rpm), secondly gas flow rate (1–2 vvm) and thirdly gas oxygen content (21–100 %).

A DO spike was used to identify complete consumption of glycerol and initiate a concentrated glycerol feed (containing 714 g/l glycerol, 50 g/l yeast extract), at 3.6 ml/l/h unless otherwise stated. dAb formation was induced when the biomass concentration had reached a level corresponding to OD_600_ = 80 ± 2, by addition of IPTG to a final concentration of 250 μM. Real time values of pH, dissolved oxygen, agitation speed, temperature, air-flow rate, oxygen percentage, oxygen uptake rate and carbon dioxide evolution rate were recorded automatically by the bioreactor software (DASGIP Control, Julich, Germany). Each set of experiments was run in parallel using duplicate fermentations and a commonly prepared inoculum.

Variations in the above standard fermentation protocol included the following: concentrated glycerol feed rates ranging from 3.0 to 4.8 ml/l/h; use of continuous addition of 125 mM EDTA (pH 7) and 125 mM EDTA 7.5 M urea (pH 7) initiated at ~15 h post-induction at 5 ml/l/h; Tween 20 addition at 15 h post-induction at 0.5 ml/l/h; batch addition of PEI (40 ml of 6.25 % *w/v*, giving at 23 h post-induction a final PEI concentration of 0.25 % (*w/v*); continuous feed of 6.25 % *w/v* PEI, solution initiated at 15 h post-induction at feed rates of 1.5 and 2.5 ml/l/h. The PEI used is branched of molecular weight 750 kDa (BASF, Ludwigshafen, Germany).

### Analytical methods

The dAb product concentration was measured using protein A affinity chromatography (HPLC Agilent 1200, Agilent Technologies UK Ltd., West Lothian, UK, fitted with a 1-ml HiTrap MabSelectR Xtra, GE Healthcare Life Sciences, Buckinghamshire, UK). Loading and equilibration were performed using a 0.1-M PBS buffer at pH 7.2. Samples were diluted appropriately in equilibration buffer and filtered using 0.22-μm PVDF syringe filter. Elution was performed using a 20 mM HCl. Product elution was recorded at 220 nm and peak areas integrated (Empower, Waters, Milford, USA).

Sample preparation for measurement of extracellular dAb was by centrifugation of 1-ml broth at 24,200 × *g* for 10 min and the supernatant was 0.2-μm filtered. For intracellular dAb, centrifuged pellet was re-suspended to 1 ml with 50 mM Tris pH 8, subjected to four freeze-thaw cycles (freezing in dry ice followed by incubation in a dry bath for 5 min at 37 °C) and two freeze-sonication cycles (freezing in dry ice followed by sonication for 15 min) using a sonication bath (Camsonix C275, Elma Electronic GmbH, Singen, Germany). The sonicated samples were centrifuged at 24,200 × *g* for 10 min and the cell lysate recovered and 0.2-μm filtered. The intracellular dAb extracted was tested for the presence of the signal peptide sequence for periplasmic secretion (in-house coupled size exclusion chromatography - HPLC/Mass Spec analysis method, Water Corp., Hertforshire, UK). Total dAb was by summation of intracellular and extracellular measurements.

The viscosity measurements were carried out using a cup-and-bob rheometer (Brookfield DV-2+ viscometer fitted with spindle 40, Brookfield Engineering Laboratories, MA), exposing 0.5 ml of cell broth to shear rates of 37 to 1200/s in seven increments with 30-s hold at each increment, for increasing and decreasing shear sweeps at 23 °C.

The concentration of the double-stranded DNA in the extracellular environment was determined using fluorometry (Qubit 2.0 Fluorometer, Invitrogen, Carlsbad, CA, USA). Sample preparation was as described above for dAb, but without the 0.2-μm filtration step.

Cell culture analysis was by (a) optical density at 600 nm; (b) gravimetrically as dry cell weight (DCW) using 1-ml samples centrifuged at 24,200 × *g* for 10 min and the recovered pellets washed twice in distilled water and then dried to constant weight in an oven at 105 °C for 24 h; (c) capacitance was measured online at 1000 kHz (Aber Instruments, Aberystwyth, UK).

The endotoxin levels in the broth supernatant were measured using a commercial portable test system with disposable cartridges containing Limulus amebocyte lysate (LAL) following manufacturer’s instructions (Charles Rivers Laboratories, Charleston, SC, USA).

The concentration of the host cell proteins (HCP) in the broth supernatant was measured using an in-house *E. coli* anti-HCP sandwich ELISA method (GlaxoSmithKline, Stevenage, UK).

## Results

**Fig. 1:**
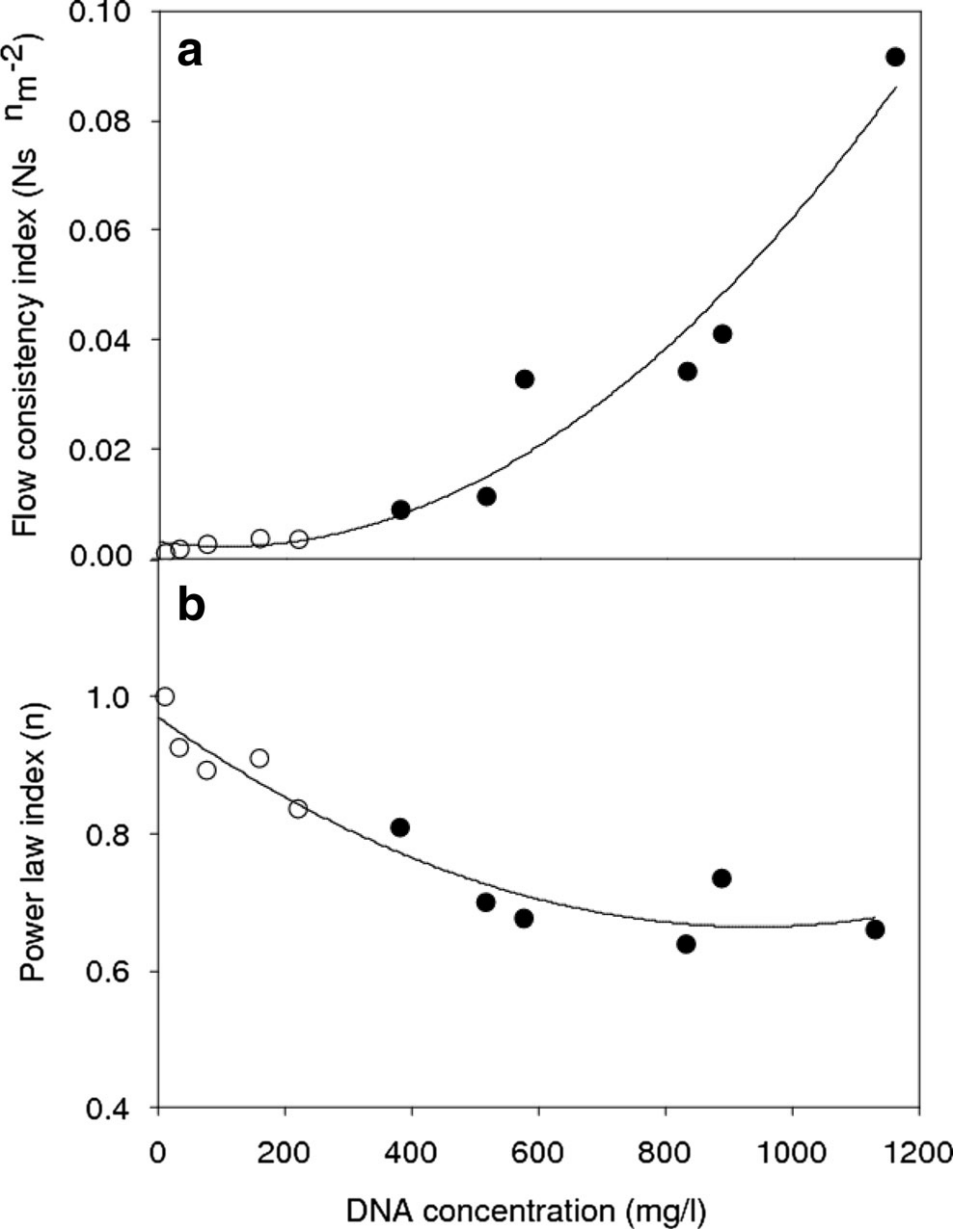
Rheological properties of *E. coli* fermentation broths as a function of extracellular DNA concentration. *Open circles* pre-induction 0–20 h, cell concentration 0 to 30 g dwt/l; *Filled circles* post-induction 20–65 h, cell concentration 30 to 40 g dwt/l. The rheological data is for power law fits, *τ* = kγ^n^, (*R* ^2^ = 0.99), *γ* = 37 to 1200/s for 30-s sweeps of both increasing and decreasing shear (no significant difference). For [DNA] >1000 mg/ml, some samples appeared to exhibit a high apparent yield stress and did not shear. Temperature = 23 °C

Figure 1 characterises the rheological properties of a range of suspensions, including lysed cell broths, as a function of the soluble DNA concentration. All solutions show characteristic pseudoplastic flow behaviour which is time independent and fully reversible. Power law expressions adequately describe the data obtained and the consistency index (equivalent to the apparent viscosity at shear rate of 1/s) gives an indication of the challenge which will be faced when trying to clarify by centrifugation. A consistency index of ~0.03 Ns^n^/m^2^ obtained at a DNA concentration of 600 mg/l is taken as the limit defining challenging centrifugation (Voulgaris et al., to be published). At this level of release, there was also visual evidence of poor fermentation mixing conditions, e.g. appearance of large air bubbles.

**Fig. 2:**
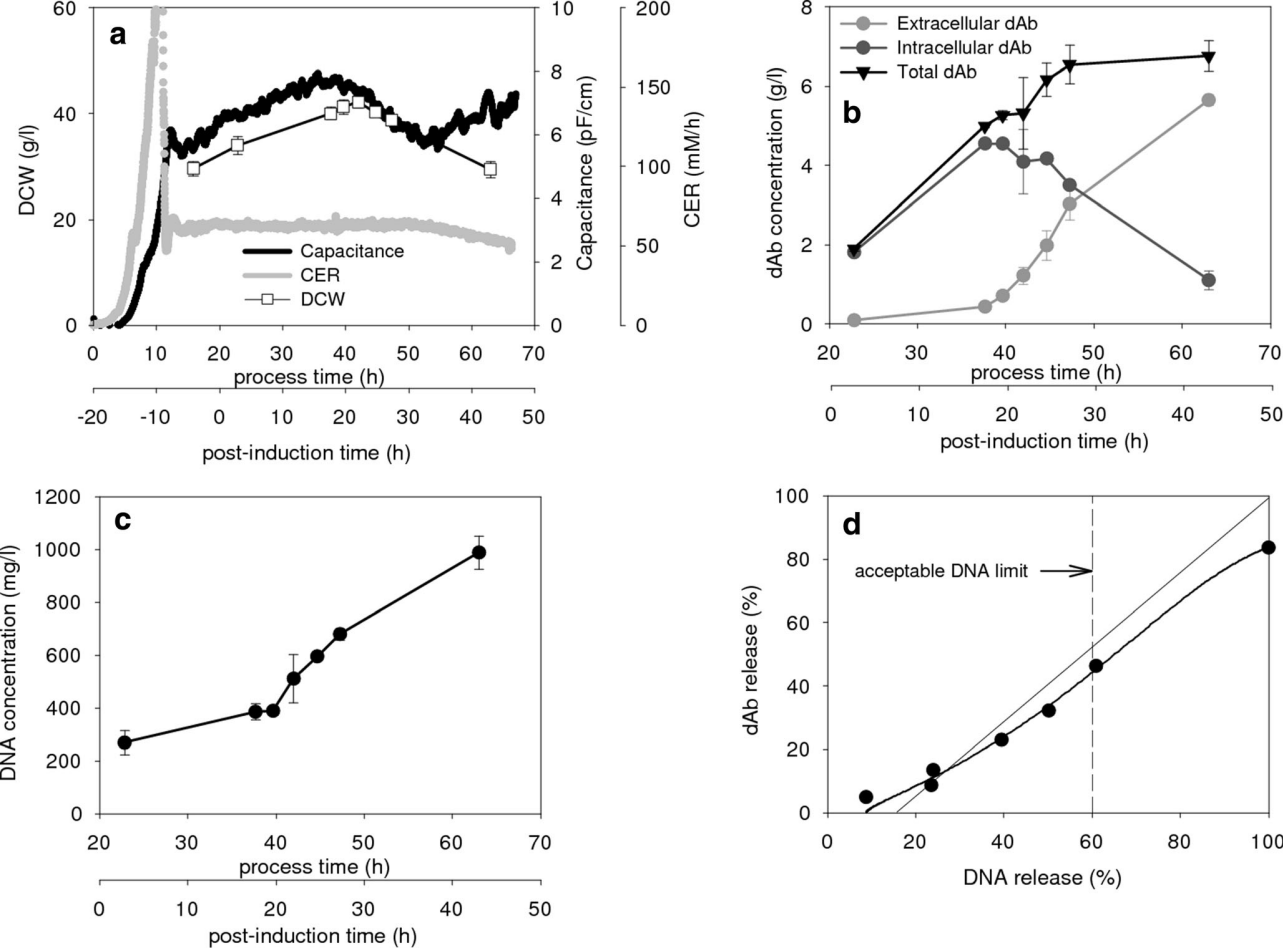
Formation of dAb fermentation and construction of parity plot relating dAb and DNA release. **a** Profile of dry cell weight, capacitance, and carbon dioxide evolution rate. **b** Profiles of extracellular, intracellular and total dAb. **c** Profiles of DNA release to the extracellular space. **d** Parity plot comparing release into extracellular environment of dAb and DNA. One hundred percent DNA release = 0.031* maximum cell DCW (Neidhardt and Umbarger 1996). Construction: parity line (*solid line*) is line of equal dAb and DNA release from induction (0 % dAb, 17 % DNA); the DNA release boundary (*dashed line*) refers to the upper limit beyond which the rheological proteins of the broth are such that clarification by centrifugation is deemed to be overly challenging—see text

Figure 2 provides an example of the standard fermentation used in this study. The post-induction time will be used to follow fermentation progression. Intact cell concentration as recorded by capacitance, peaks at 15 h; this is followed soon after by a peak in dry cell weight (includes dying cells and cell debris (Sonnleitner 2013) and a drop in cell respiration (lower CER) at ~30 h (Fig. 2a). This profile is matched by the onset of a significant rise in extracellular domain antibody (dAb) and parallel reduction in intracellular dAb at 15 h and the attainment of a maximum level of total dAb at 30 h (Fig. 2b). No evidence was found of intracellular dAb containing a signal peptide sequence; hence, from here on, intracellular dAb refers to processed dAb available in the periplasmic space. The release of DNA (Fig. 2c) increases sharply at the onset of a reduction in DCW and appears to parallel the release of dAb except for an initial high level of DNA present at the onset of induction.

A parity plot (Fig. 2d) follows dAb release with DNA release. The parity line follows the release of dAb and DNA from the initial value at induction to the maximum available on complete disruption of whole cells and cell ghosts. The maximum values are by measurement of dAb and for DNA by estimation from the maximum cell dry weight; this is due to difficulties in the release of all DNA in an intact form for measurement. The observed upper limit of 80 % of maximum available dAb might be defined as the target to be achieved by release during fermentation, i.e. by cell autolysis. An acceptable DNA release boundary of 60 % is drawn in; i.e. equivalent to 600 mg/l (see Fig. 1). At this DNA release limit, only ~40 % of the dAb has been released and the challenge is to find ways of moving above the parity line to achieve high dAb recovery with acceptable limited DNA release.

**Fig. 3:**
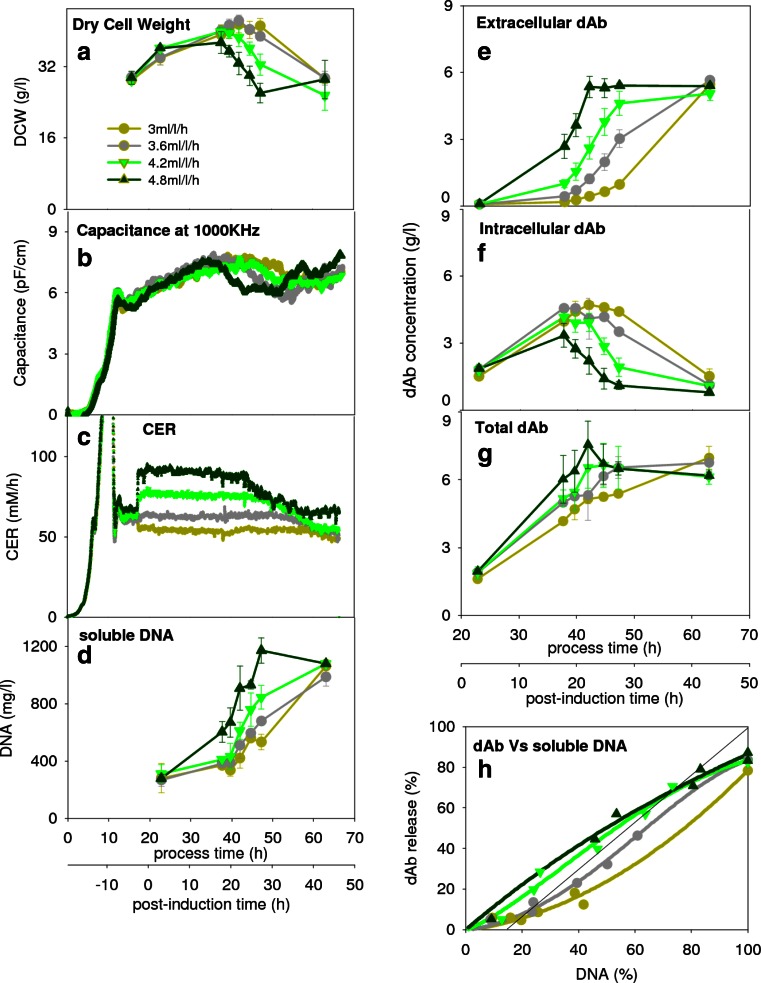
Effect of carbon source feed rate (see *inset*) on product formation and comparative product versus DNA release. Time profiles of **a** dry cell weight, **b** capacitance at 1000 kHz, **c** carbon dioxide evolution rate, **d** DNA release to the extracellular space, **e** extracellular dAb concentration, **f** intracellular dAb concentration, **g** total dAb concentration and DNA release to the extracellular space and **h** parity plot comparing release into extracellular environment of both dAb and DNA. The CER values decline at 38, 32, 28 and 23 h post-induction for the 3.0, 3.6, 4.2 and 4.8 ml/l/h feed rates, respectively

The effect of changing cell growth rate by adjustment of the carbon source feed rate after induction and the resulting balance of dAb to nucleic acid release is explored in Fig. 3. The range of feed rates chosen was based on a high-end value to avoid excess glycerol accumulation and a low-end value below which fermentation exceeds 50 h post-induction. Higher feed rates appear to result initially in small (DCW basis) or no (capacitance basis) increases in biomass levels and then earlier lysis and ultimately lower biomass levels (Fig. 3a, b). The CER values (Fig. 2c) reflect the increased biomass activity (up to 20 h) and the earlier onset of lysis as the feed rate is increased. As before, the DNA release (Fig. 3d) reflects the extent of cell lysis. The final total intracellular and extracellular dAb levels are largely unaffected by the choice of feed rate (Fig. 3e–g) but the higher rates of dAb formation at greater feed rates reflects the higher biomass activity (Fig. 3c, g), and the earlier release into the extracellular space at greater feed rates reflects the earlier onset of cell lysis (Fig. 3a, b, e). The resultant parity plot (Fig. 3h) shows that the greater the feed rate, the higher the extent of release of dAb compared to DNA. The improvement achieved increases the dAb yield from ~35 to ~58 % for 60 % DNA release. However, if the target is near complete recovery of dAb, then it will, in all cases, be accompanied by ~100 % DNA release.

**Fig. 4:**
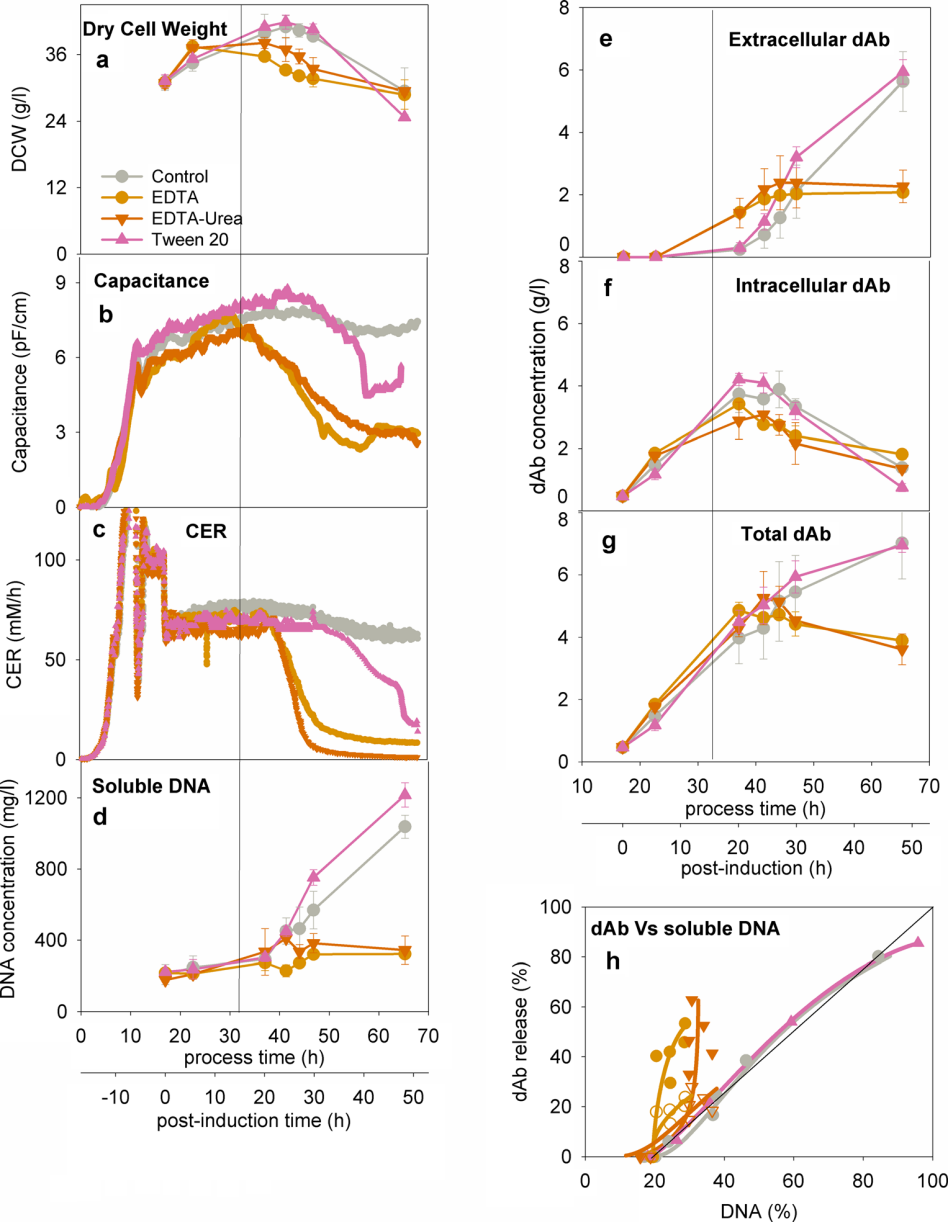
Effect of alternative release strategies on dAb formation and relative extracellular concentrations of dAb and DNA—see Fig. 3 for key. For Fig. 3h, closed points are for % dAb release against maximum level achieved in EDTA or EDTA-urea fermentations; open points are for % dAb release against maximum as obtained in control. Operation was as for the control culture with a feed rate of 3.6 ml/l/h. Cultures were treated with (i) EDTA 125 mM at 5 ml/l/h to final [EDTA] of 18 mM, (ii) with EDTA 125 mM urea 7.5 M at 5 ml/l/h to final [EDTA] of 18 mM and [urea] of 1 M, (iii) Tween 20 incrementally over 23 h (done in a controlled way to avoid foaming) to a final concentration of 20 ml/l. The line at 35 h process time signifies the start of the EDTA and EDTA-urea feed

The use of cell permeabilising agents is explored in Fig. 4. These agents were selected from literature (see Introduction) and the concentrations used are based on laboratory-based investigations of ranges of final concentrations of 15 to 75 mM EDTA, 0.5 to 2 M urea and 10 to 20 ml/l Tween 20 (results not shown here). The use of EDTA or EDTA-urea results in a decrease in biomass as evidenced by lower values of dry cell weight (Fig. 4a) and capacitance (Fig. 4b) and reduced CER values (Fig. 4c). The resultant effect is low released DNA levels and total dAb levels (Fig. 4d, g) but with a significant proportion of the dAb appearing extracellularly (Fig. 4e) evidently as a result of the increased cell wall permeabilisation. As a result, there is an overall ~3-fold increase in the ratio of the final dAb to the final DNA release. This improvement is largely due to the reduced DNA levels and indeed, recalibration of dAb release against the level of dAb formed in the control brings all the EDTA and EDTA-urea data points back to the parity line. The use of Tween resulted in an earlier onset than the control in loss of active biomass (Fig. 4a–c) but little difference in the released DNA of dAb profiles (Fig. 4d–g) resulting in no improvement in the ratio of dAb to DNA release (Fig. 4h). Also, high levels of foaming were experienced. These strategies were not pursued further as the small-scale screening trials indicated little room for improvement with these particular reagents.

**Fig. 5:**
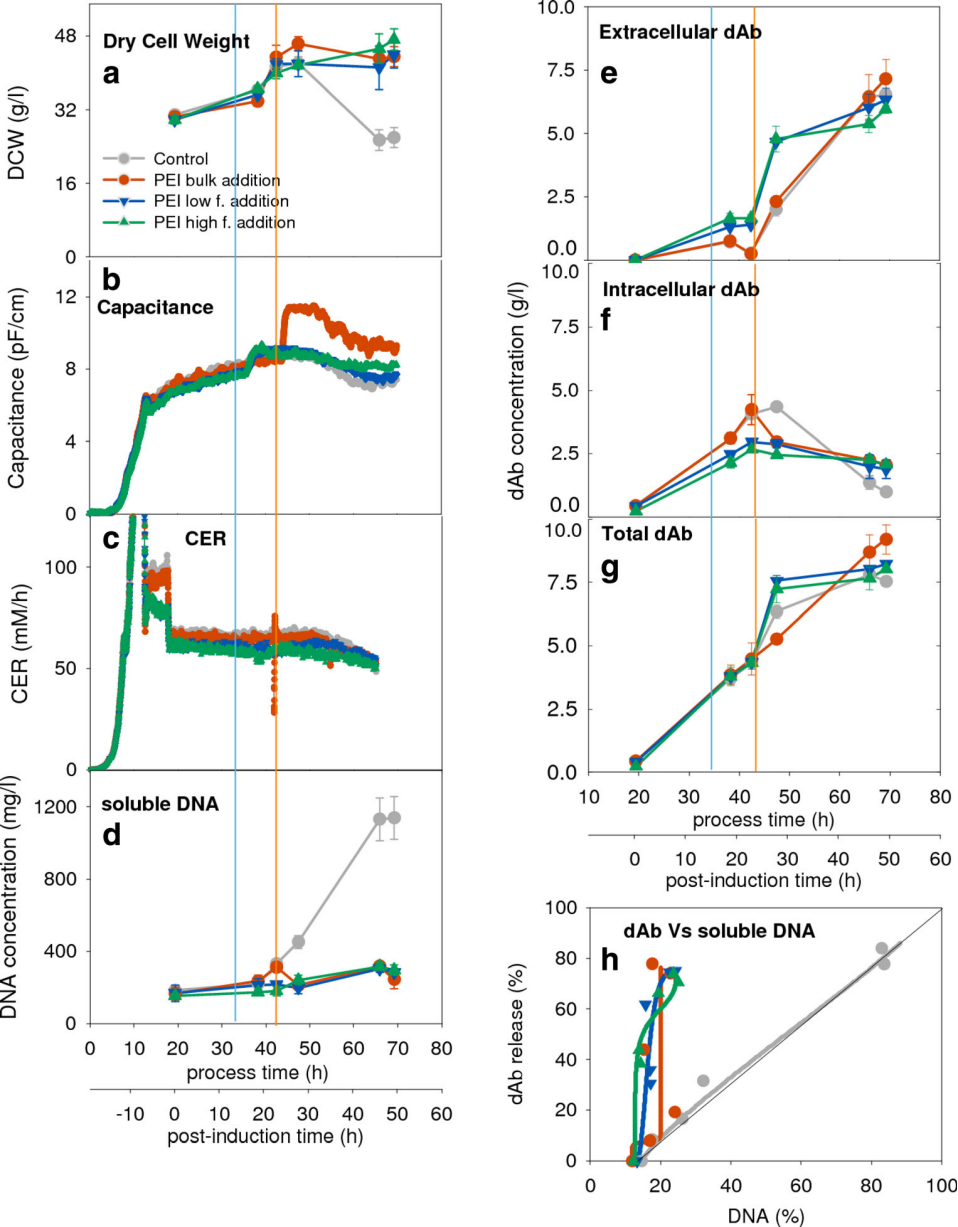
Effect of alternative PEI feed strategies on product formation and relative extracellular concentrations of product and DNA—see Fig. 3 for the key. Operation was as for the control culture with a feed rate of 3.6 ml/l/h. Cultures were treated with PEI to a final concentration of 3 and 5 g/l, respectively, with a low concentration continuous feed of 1.5 ml/l/h for 32 h and a high concentration of 5 g/l continuous feed at 2.5 ml/l/h for 32 h. The vertical lines at 32 and 42 h process time signifying the onset of the PEI feeds and PEI bulk additions respectively

The use of PEI as both a potential release reagent and a DNA precipitation reagent is studied using three feeding strategies, namely in bulk as a single shot and continuously at low or high feed rate (Fig. 5). The choice of feeding concentration and quantity was to ensure sufficient PEI was present to precipitate the DNA to a maximum extent, and not so much as to disturb the pH balance of the culture. In all cases, the use of PEI resulted in an increased dry cell weight although evidently, this measurement is compromised by the presence of PEI-precipitated material. The capacitance and CER readings (Fig. 5b, c) indicate the PEI had little effect on active biomass. The increased capacitance observed (in duplicate) when using bulk PEI addition might be due to liquid entrapped within cell flocs formed relatively early in the induction phase by using this method of addition. To note, there is a small but significant increase in capacitance for continuous PEI feeds (not observable in graphs shown); this is probably due to the same reason of floc formation. The level of soluble DNA remains low in the presence of PEI (Fig. 5d). For all the PEI feed strategies tested, the proportion of dAb released is greater than for the control as might be expected from the permeabilising effect of PEI. Within this limited study, there is some evidence for greater release with earlier onset of PEI addition and little evidence for benefits of use of higher concentration of PEI feed. Little effect is noted for any PEI addition strategy on the resultant overall dAb level and location at the end of the fermentation (Fig. 5e–g). The result is an overall ~ 4-fold increase in the ratio of dAb to DNA release which approaches the maximum possible dAb which can be released by autolysis, e.g. in comparison with the upper limit shown in Fig. 2d. Since it is advantageous to avoid overdosing with PEI (carry through of excess soluble PEI might affect performance of later purification stages) the low concentration PEI feed strategy was taken forward for further study. The choice of PEI used, i.e. branched 750 kDa, is discussed later. Separate fermentations, results not shown here, using branched 25 kDa PEI, with the same continuous low feed rate administration as used for the 750 kDa, resulted in high levels of early cell death—to be discussed later.

**Fig. 6:**
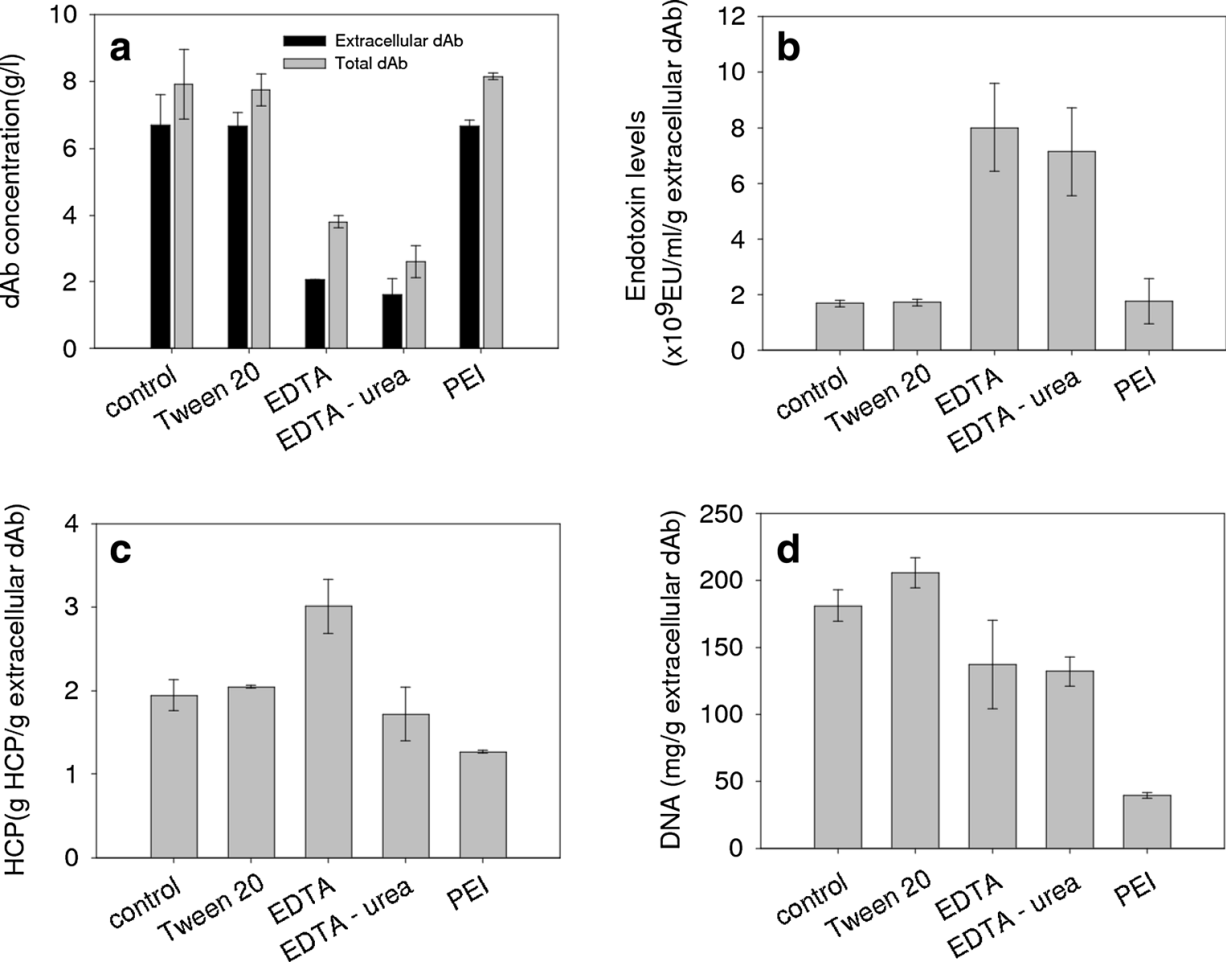
Impact of different cell release strategies on **a** extracellular and total dAb, and per unit of extracellular dAb **b** endotoxin, **c** HCP and **d** soluble DNA. All fermentation carried out for 45 h post induction and reached a final cell density of ~25–30 g dwt/l. The release agents were applied as described in Figs. 4 and 5 (for the PEI treatment the continuous low feed was used)

Figure 6 summarises the performance of the various release agents used in Figs. 4 and 5 in terms of the quality of the final broth obtained. The dAb concentration at harvest remains similar for the control-, Tween- and PEI-treated cultures while the EDTA and EDTA-urea have significantly lower (~3-fold) total and extracellular values. Figure 6b shows a ~3-fold increase in endotoxin/extracellular dAb ratios for the EDTA and EDTA-urea cultures. The PEI-treated cultures had endotoxin levels similar to the control and Tween 20 cultures. The HCP released or remaining in broth is not affected by Tween (compared with control) but is reduced ~2-fold for EDTA, ~3-fold for EDTA-urea and ~1.3 fold for PEI. Figure 6c shows that HCP/extracellular dAb ratios were significantly higher for the EDTA but not the EDTA-urea-treated cultures. PEI-treated cultures exhibited decreased levels of HCP/extracellular dAb ratio which can either be attributed to lower cell lysis during the PEI treatment or to precipitation of some of these proteins by the polycation. Finally, soluble DNA in the fermentation broth is ~5-fold lower for the PEI culture compared to control and Tween 20 cultures. EDTA- and EDTA-urea-treated cultures exhibited marginally lower DNA/extracellular dAb ratios due to extracellular dAb being threefold lower.

**Fig. 7:**
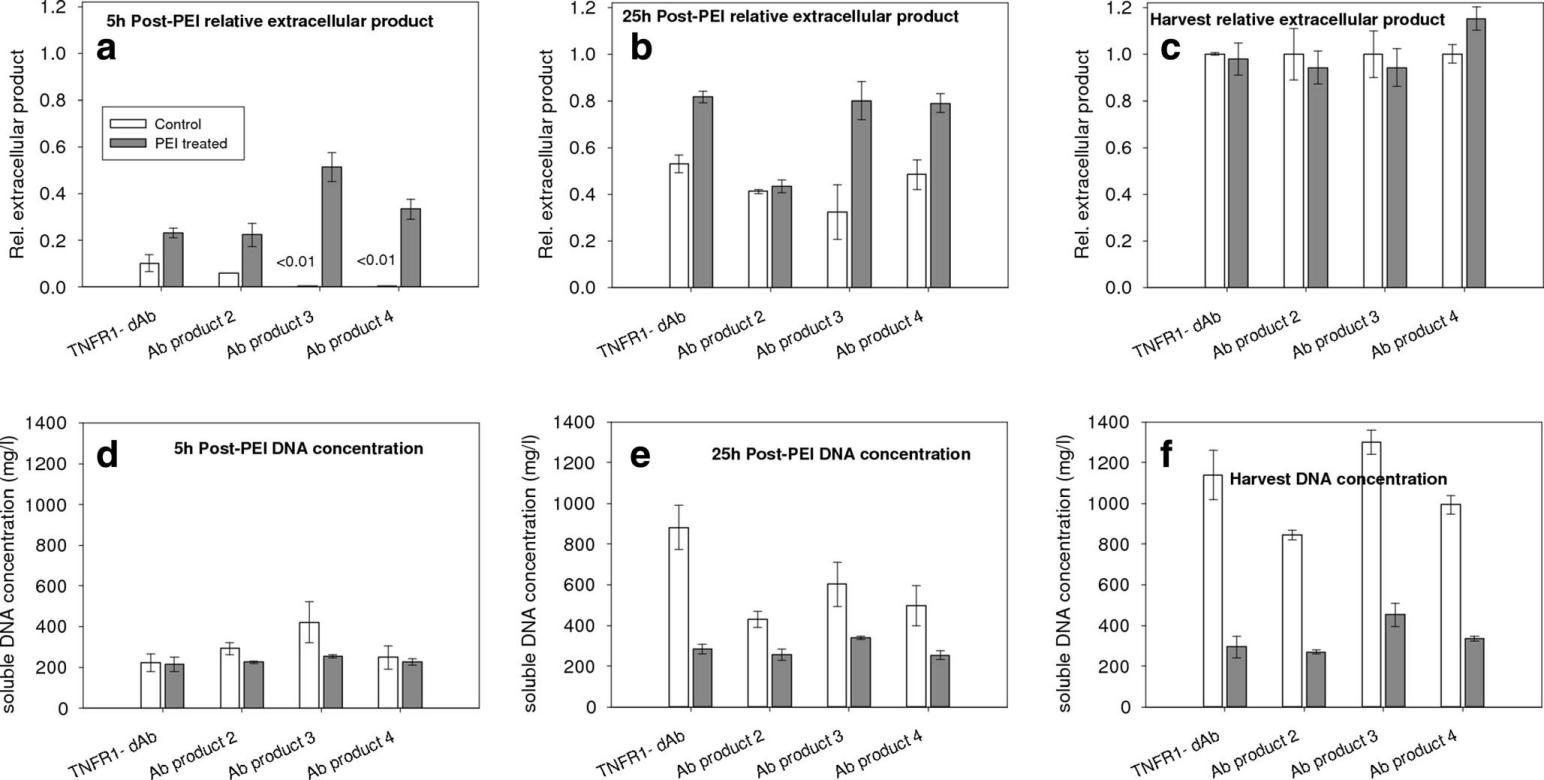
The effect of PEI treatment (1.5 ml/l/h) on product release and soluble DNA concentration for four different recombinant antibody fragment producing constructs (including for the dAb presented in Figs. 2–6). Relative recoveries are given for 5 and 25 h after the onset of the PEI treatment as well as at the harvest (50 h post-induction, 46 h after start of PEI treatment). The relative extracellular product is the proportional increase in released dAb (dAb released using PEI/dAb released at harvest without PEI). Application of PEI started at ~4 h after the induction of the recombinant antibody product

Figure 7 shows the effect of the PEI treatment on the recombinant production of different antibody molecules in *E. coli* culture. Antibody products 2, 3 and 4 are of MW ~25, 14 and 17 kDa. respectively. Figure 7a shows significantly increased product release with the PEI application while as time passes the difference between PEI treated and control culture becomes less pronounced (Fig. 7b) especially for the larger antibody product 2 and, at the end of the process, the levels of extracellular product are almost identical (Fig. 7c). The difference of concentration of the extracellular soluble nucleic acids between the control- and PEI-treated cultures is increased with time (Fig. 7d–f). As cells are gradually releasing more nucleic acids, PEI precipitates them retaining the concentration below 400 mg/l, i.e. below critical level of 600 mg/l.

## Discussion

One effect of increasing lysis during *E. coli* fermentation is the release of nucleic acids leading to considerable increases in broth viscosity (Nesbeth et al. 2012). This can lead to poorer transport processes during fermentation especially at industrial scale (Gabelle et al. 2011), e.g. more challenging heat and mass transfer and poorer mixing albeit at a time when cell growth is largely complete so issues of nutrient supply and metabolic heat removal are less demanding. The broth viscosity is also an important factor affecting downstream processes and in particular, clarification by centrifugation (Balasundaram et al. 2009) and filtration (Davies et al. 2000). For *E. coli* expression systems even where product is secreted to the periplasmic space, release of product from the cell is accompanied by equivalent release of DNA from the cell (Fig. 2). However, it is only necessary to limit DNA release to ~60 % of total DNA while achieving near complete product release to have a broth acceptable for processing. Hence, the challenge is to manipulate fermentation conditions such that the cells preferentially release the product or the released DNA at least in part is taken out of solution.

The effect of varying cell growth rate has been shown to change the outer membrane phospholipid composition for *E. coli*, increasing membrane fluidity and allowing periplasmic material to be released (Shokri et al. 2002). Also, for *E. coli*, higher growth rates lead to reduced levels of the outer membrane proteins OmpF and LamB which results in greater membrane permeability (Bäcklund et al. 2008). These observations are consistent with the results reported in Fig. 3, where for a limit of 60 % DNA release, the effect of greater feed rate is to increase the dAb release from 35 to 58 %. However, for near-complete dAb release, it is necessary to allow full cell lysis and hence, high levels of DNA release. Consequently, there is a need to explore ways to change the relationship between dAb and DNA release during fermentation if opportunities are to be realised for earlier harvest and direct recovery of dAb without further pretreatment.

EDTA is known to permeabilise cell membranes and hence aid the release of proteins (Jalalirad 2013). For example, use of EDTA in *E. coli* culture was successful in increasing the levels of two recombinant enzymes, although a biomass drop of 22 % was observed (Luo et al. 2014). The permeabilisation is probably due to the interaction of EDTA with the membrane stabilising cations such as Mg^2+^ and Ca^2+^. These counterions counteract the negative repulsive charges of the adjacent LPS molecules which link them electrostatically, and stabilise the structure (Polissi and Sperandeo 2014).The damage of the LPS leaflet compromises the integrity of the OM (Vaara 1992). However, the use of EDTA proves to be almost immediately toxic to the cells (Fig. 4b, c). This might be due to the sequestering of Fe^3+^ (Komárek et al. 2007), thereby depleting iron containing proteins such as cytochrome and ferredoxin, which are part of the electron transport chain and hence reducing respiration. Moreover, iron is required in other processes such as in amino acid and pyrimidine biosynthesis, DNA synthesis and in superoxide radical protection (Earhart 1996). However, when the iron concentration exceeds cellular requirements, it is detrimental to the cell growth as it promotes Fenton reaction causing DNA, protein, and lipid damage (Imlay 2013). The use of chelating agents such as EDTA should reduce such effects.

The EDTA treatment led to significantly lower amounts of soluble DNA present (Fig. 4c). This may be partly attributed to the lower biomass levels. The presence of EDTA protects DNA from nuclease attack as it sequesters Mg^2+^ which acts as cofactor (Yagi et al. 1996). However, such cation chelation may destabilise the double-stranded state of DNA and lead to its denaturation to a single-stranded form which is not detected by the fluorometric method used in this paper (Sedlackova et al. 2013). The capacitance data (Fig. 4b) suggest that there is a sharp drop of the concentration of cells with intact membranes, so it is unlikely the cells have entered a metabolically inactive state where the cells remain intact.

The overall result is an apparent improvement in the relationship of dAb to DNA release (Fig. 4h) which, when the lower dAb levels are taken into account, is effectively no improvement in release selectivity over the control. The effects of decreases in broth viscosity on flow were apparent (not shown here) probably due to reduced DNA levels; this is in contrast to increased viscosity in the case of an *Aspergillus niger* culture due to increased local hydrophobic interactions between the mycelia (Berkman-Dik et al. 1992). The EDTA-treated cultures had significantly higher endotoxin level per product (Fig. 6b) again due to the EDTA interaction with the membrane-stabilising cations destabilising the OM and releasing lipopolysaccharide molecules. The HCP levels are significantly higher for the EDTA-treated cultures (Fig. 6c) probably due to the extensive cell lysis as discussed earlier.

The use of urea in combination with EDTA was studied to explore if the chaotropic effect of urea on membrane lipids (Jalalirad 2013) can improve dAb recovery. However, since EDTA had so significantly reduced cell growth (Fig. 4a–c) and remaining intracellular dAb (Fig. 4f), little effect could be observed. The use of urea did lead to significantly lower HCP levels present (Fig. 6) possibly due to denaturation of some proteins leading to their removal by precipitation (Bennion and Daggett 2003).

The non-ionic detergent Tween has been shown to permeabilise the cell membrane by solubilising and removing the OM lipids (Jamur and Oliver 2010). An almost ~4-fold increase in the activity of a recombinant enzyme from 0.6 to 2.2 % of the total activity has been documented when Tween was applied in *E. coli* shake flasks (Luo et al. 2014). While Tween had little effect on the cell growth and dAb formation in the studies reported here, little to no effect was also observed on the dAb release (Fig. 4h) or on the overall contaminant profile (Fig. 6).

The use of PEI to help process harvested fermentation broths is well known (Chatel et al. 2014b; Salt et al. 1995). Here, we are concerned with the use of PEI during the fermentation itself and the impact on live cells. For example, for *Salmonella typhirnurium,* the effect of PEI is to redistribute phospholipids from the inner to the outer layer and to enlarge the OM surface area (Azevedo et al. 2014; Helander et al. 1998); for human cell lines, PEI induces membrane degradation and initiates apoptosis (Moghimi et al. 2005). For *E. coli,* it has been suggested that PEI permeabilises the OM by intercalation (Alakomi et al. 2006). This, with the observation that PEI does not cause significant disruption of the cytoplasmic membrane, suggests that the cells remain viable while becoming permeable (Gibney et al. 2012). This result is apparent in Fig. 5 with enhanced leakage of dAb to the broth (Fig. 5e) while the cells remain viable (Fig. 5a–c). The method of administration of the PEI seems to have a significant effect on the dAb release but this needs a more detailed study to characterise sufficiently.

The impact of PEI on the LPS concentration in the culture is complex. It has been observed that PEI do not release LPS when it permeabilises the outer membrane (Alakomi et al. 2006). Similarly for *Salmonella typhirnurium,* while significant LPS release occurs after treatment of cells with permeabilisers such as EDTA, PEI causes no release (Helander et al. 1997). However, if LPS is released, then at neutral pHs, i.e. as during the fermentation, its partially phosphorylated carbohydrate residues will have a net negative charge (Petsch et al. 1998) which should lead their removal by precipitation. As such, PEI has been used for endotoxin removal from systems as diverse as whole blood (Mitzner et al. 1993), model endotoxin–protein mixtures and crude *E. coli* lysates (Hanora et al. 2005). The low levels of LPS release observed when using PEI in *E. coli* fermentations (Fig. 6) might either be the result of little to no LPS release or LPS precipitation or both.

As with LPS, it is unclear if the decreased HCP and DNA levels (Fig. 6) are due to reduced lysis or due to precipitation or a combination of both. PEI is very potent at binding and precipitating negatively charged macromolecules such as DNA, RNA, and proteins bound to DNA or RNA, as well as acidic proteins (Mazzaferro et al. 2010). Similar results of DNA and lipid reduction, due to removal by PEI precipitation, have been reported in culture homogenates (Salt et al. 1995). Likewise, PEI has been shown to precipitate colloidal protein (Salt et al. 1995) and this might be the mechanism of HCP reduction noted here. At post-induction times of ~50 h, the indication is that the cells in the presence of PEI have started to lyse but probably not to the same extent as for the control without PEI (Fig. 5a–c). Here, the PEI will be acting as a precipitating agent while earlier in the fermentation, it may have promoted the dAb release with reduced DNA or other intracellular component release. The analytical methods used here were not able to distinguish between the two mechanisms as there is a need for sample preparation to fractionate the precipitate from the cells and cell ghosts.

Evidently, there may be other effects of PEI on cell metabolism to be taken into account. For example, PEI inhibits biofilm formation in *Staphylococcus* and *Acinitobacter* species (Azevedo et al. 2014) and in *E. coli* (Alakomi et al. 2006) which might improve bioreactor performance by avoiding energy- and nutrient-wasteful biofilm synthesis and improving mass and heat transfer (Sung et al. 2006). PEI enhances antibiotic penetration (Khalil et al. 2008) and hence might enhance the segregation stability of the cultivating culture by avoiding plasmid loss. It is also possible that PEI-related early release of dAb from the periplasm will reduce pressure on the cell physiology.

The choice of PEI form and molecular weight might offer an important means for optimising its use during fermentation. In this study, the choice was made primarily based on the ability of PEI to precipitate selectively released cell components. High molecular weight branched PEI was used due to its high hydrophobicity which results in a reduced number of polymer chains available to interact with the bacterial cell membrane and hence lower toxicity (Gibney et al. 2012). Also, the high branched 750 kDa PEI will not pass through the OM due to its larger structure and therefore will destabilise only the OM. In contrast, lower molecular weight and linear PEI will be less likely to be trapped by the outer membrane anionic LPS and peptidoglycan (Gibney et al. 2012; Takahashi et al. 2013) and hence, there is a higher possibility of destabilisation or destruction of the inner membrane (e.g. as noted in the “Results” use of 25 kDa PEI led to significant cell death).

Figure 7 demonstrates the application of use of PEI during fermentation for three other cell constructs preparing different dAb-related proteins. In all cases, the PEI has an immediate positive effect on the release of the product, the ratio of product to soluble DNA is enhanced, the final product titre is unchanged compared to the control and, when full release of the final product is achieved, the level of soluble DNA remaining is well below the limit to yield broths suitable for clarification by continuous centrifugation at manufacturing scale.

Some key conclusions are as follows:

(i) The use of increased feed rates to enhance cell growth rate not only helps reduce fermentation time but gives a small preferential release of the dAb compared with DNA. However, this advantage is not realised at high levels of dAb release as might be required in manufacture.
(ii) The use of EDTA probably helps permeabilise the cells, but it prevents cell growth; hence precluding its use to after fermentation is completed rather than during fermentation.
(iii) The use of PEI during fermentation does not cause any adverse effects on cell growth or on dAb formation and does enhance the release into the broth of the dAb. Furthermore, the presence of PEI leads to reduced levels of soluble HCP, LPS and DNA, the last of which is predicted to result in a broth which will be easier to process by continuous centrifugal clarification at large scale.
